# Lumbar plain radiograph is not reliable to identify lumbosacral transitional vertebra types according to Castellvi classification principle

**DOI:** 10.1186/s12891-020-03358-3

**Published:** 2020-05-29

**Authors:** Lisheng Hou, Xuedong Bai, Haifeng Li, Tianjun Gao, Wei Li, Tianyong Wen, Qing He, Dike Ruan, Lijing Shi, Wei Bing

**Affiliations:** 1 Department of Orthopedic Surgery, the sixth Medical Center of PLA Army General Hospital, NO.6 Fucheng Road, Beijing, 100048 China; 2 Department of Radiology, The sixth Medical Center of PLA Army General Hospital, Beijing, China; 3 Department of Computer Center, The sixth Medical Center of PLA Army General Hospital, Beijing, China

**Keywords:** Lumbosacral transitional vertebra, Lumbar plain radiograph, Castellvi classification, Coronal reconstructed CT image, Misclassification

## Abstract

**Background:**

The anteroposterior view of the lumbar plain radiograph (AP-LPR) was chosen as the original and first radiographic tool to determine and classify lumbosacral transitional vertebra with morphological abnormality (MA-LSTV) according to the Castellvi classification. However, recent studies found that AP-LPR might not be sufficient to detect or classify MA-LSTV correctly. The present study aims to verify the reliability of AP-LPR on detecting and classifying MA-LSTV types, taking coronal reconstructed CT images (CT-CRIs) as the gold criteria.

**Methods:**

Patients with suspected MA-LSTVs determined by AP-LPR were initially enrolled. Among them, those who received CT-CRIs were formally enrolled to verify the sensitivity of AP-LPR on detecting and classifying MA-LSTV types according to the Castellvi classification principle.

**Results:**

A total of 298 cases were initially enrolled as suspected MA-LSTV, among which 91 cases who received CT-CRIs were enrolled into the final study group. All suspected MA-LSTVs were verified to be real MA-LSTVs by CT-CRIs. However, 35.2% of the suspected MA-LSTV types judged by AP-LPR were not consistent with the final types judged by CT-CRIs. Two suspected type IIIa and 20 suspected type IIIb MA-LSTVs were verified to be true, while 9 of 39 suspected type IIa, 9 and 3 of 17 suspected type IIb, and 11 of 13 suspected type IV MA-LSTVs were verified to truly be type IIIa, IIIb, IV and IIIb MA-LSTVs by CT-CRIs, respectively. Incomplete joint-like structure (JLS) or bony union structure (BUS) and remnants of sclerotic band (RSB) between the transverse process (TP) and sacrum were considered to be the main reasons for misclassification.

**Conclusion:**

Although AP-LPR could correctly detect MA-LSTV, it could not give accurate type classification. CT-CRIs could provide detailed information between the TP and sacrum area and could be taken as the gold standard to detect and classify MA-LSTV.

## Background

Lumbosacral transitional vertebra (LSTV) is regarded as a congenital anomaly that has morphologic characteristics mixed between those of sacral and lumbar vertebrae, including lumbarization of the highest sacral segment (S_1_) and sacralization of the most inferior lumbar segment (L_5_) [1, 2].

Some LSTVs exhibit obvious morphological abnormality (MA-LSTV), with or without numerical variance [3], while others exhibit only numerical variance (NV-LSTV) [4–6]. Lumbar plain radiograph (LPR) could detect MA-LSTV while misinterpret NV-LSTV as a normal spinal sequence, as the latter has insidious characteristics. Only whole spinal images could discern NV-LSTV [4–6].

Various LSTV categories exist [3–5]. The widely accepted Castellvi system is a classification of MA-LSTV based on the relationship assessment between the transverse process (TP) of suspected MA-LSTV and the sacrum on the unilateral or bilateral side(s). Four types of MA-LSTV have been classified: type I exhibits an enlarged TP measuring at least 19 mm in the craniocaudal dimension (a, unilateral; b, bilateral); type II exhibits a joint-like structure (JLS) (a, unilateral; b, bilateral) with sclerotic band(s); type III exhibits a bony union structure (BUS) (a, unilateral; b, bilateral); and type IV includes a unilateral type II transition along with a type III on the contralateral side.

Initially, Castellvi types were identified on a 30° angled anteroposterior (AP) view of the lumbar spine, which is regarded as the true AP of the lumbosacral joint (Ferguson view), aiming to decrease the radiographic overlap of the TP(s) of MA-LSTVs on the sacrum [3]. However, the Ferguson view is not routinely taken in the standard radiographic assessment of the lumbar spine, which typically includes standard AP-LPR and lateral LPR [5]. Type I MA-LSTV is not regarded as true LSTV by many specialists, as standard AP-LPR might produce false positive findings due to the overlap effect of the TP on the sacrum. Moreover, this type had little clinical significance [7]. It seems easy for AP-LPR to detect the other three MA-LSTV types for their obvious abnormal characteristics [3].

The opinion that AP-LPR could detect MA-LSTV does not mean that AP-LPR could classify MA-LSTV types accurately. Plain radiography has some inherent limitations—it frequently misses subtle or occult fractures [8] or fails to evaluate bony unions accurately [9]. Recently, some studies have pointed out that coronal MRI is superior to standard AP-LPR in detecting and classifying of MA-LSTV [5, 10]. Therefore, we hypothesize that the objective anatomic relationships between the TP of the MA-LSTV and sacrum might not always be distinguished accurately by AP-LPR.

Helical CT could provide multiplanar reconstructed CT images as desired and exhibit precise anatomic details [9]. Coronal reconstructed CT images (CT-CRIs) could provide detailed osseous anatomic structures for accurate Castellvi type identification. The present retrospective study is to verify the sensitivity and specificity of AP-LPR to detect and classify MA-LSTV types according to the Castellvi classification principle, using CT-CRIs at the bone window from a 256-slice helical CT machine as the gold standard.

## Methods

Approval for this study was obtained from the Sixth Medical Centre of PLA General Hospital Ethics Committee. The data of patients with low back pain who received AP-LPR in our hospital between June 2014 and June 2018 were extracted from the electronic database. All AP-LPRs were assessed regarding the presence of MA-LSTVs (type II to IV according to the Castellvi classification principle). The suspected MA-LSTVs judged by AP-LPRs were primarily enrolled. Among them, those who received helical CT (256-slice spiral CT, Philips) scanning with CT-CRIs of the lumbosacral region were formally enrolled into the formal study group. All the patients had CT examinations for clinical needs, rather than intentionally for this study.

Standard AP-LPRs were taken for all enrolled patients. The X-ray beam was positioned approximately 7 cm above the pubic symphysis. All images were obtained with digital radiograph (DR) equipment. For CT scanning, the individual laid on the examination bed in the supine position with the longitudinal spinal axis coincident with the machine’s longitudinal axis. Then, the patient was scanned by 256-slice spiral CT from T12 to sacrum. Images were photographed in bone detail. Raw transaxial sectional CT images were then reconstructed by the interpolation reconstruction method to get reconstructed CT images, including sagittal and coronal images at the bone window (window width: 2000, window level: 350). All images were reviewed and reconstructed at the terminal computer of picture archiving and communication system (PACS).

The finally enrolled cases were then classified into different types based on AP-LPRs and CT-CRIs, respectively, according to the Castellvi classification principle. All AP-LPRs and CT-CRIs were respectively reviewed to identify different types of MV-LSTVs by two experienced spine doctors independently. Six months later, in a second reading, the radiographs were reviewed in a random order by the same spine doctors. In the case of different opinions, two more doctors, including one professor on spine research and one senior radiologist, were collected together to draw the final conclusions.

The types of MA-LSTVs determined by CT-CRIs were used as the gold criteria and compared with the suspected types determined by AP-LPR to testify to the sensitivity of AP-LPR in the classification of MA-LSTV types.

The orientation of each JLS side of the MA-LSTV was observed on AP-LPR. Oblique LPR and raw transaxial CT images were obtained to see whether the orientation of the JLS was parallel to the horizontal line in the coronal plane, to the inferior endplate of the MA-LSTV on oblique LPR, or to the coronal line in the transaxial plane.

### Statistical analysis

Kappa statistics were used to assess the inter- and intra-observer differences for the classification of MA-LSTV types. The reliability of classification of MA-LSTV types by AP-LPR compared with the CT method was also assessed by kappa statistics.

Kappa values greater than 0.75 are defined as excellent agreement, and values below 0.50 are defined as poor agreement [11].

## Results

A total of 2026 patients who received AP-LPR were collected from the database. Among them, 298 cases, including 104 males and 94 females with an average age of 48.3 ± 7.6 years ranging from 16 to 85 years old, were suspected to be MA-LSTVs (Castellvi type II to IV) determined by AP-LPRs. The exposure settings for AP-LPRs were 80–90 kV and 20–30 mA. Among these 298 cases, 91 cases, including 43 males and 48 females with an average age of 47.5 ± 17.8 years ranging from 17 to 85 years old who received CT-CRIs of the lumbar region, were enrolled in the final study. The exposure settings for the CT scan were 100–140 kV and 100–180 mAs, and 2 mm thick slices were obtained with 1 mm reconstruction intervals. Among these 91 cases, 41 cases were associated with disc herniation, 9 with spondylolysis, 5 with degenerative spondylolisthesis, 7 with spondylolytic spondylolisthesis, and 4 with vertebral fractures.

Among these 91 finally enrolled cases, there were 7 cases of disagreement determined by AP-LPRs and 1 case of disagreement determined by CT-CRI on type classification between the two spine doctors. However, after discussion of these cases by all four doctors, clear conclusions were made without different opinions. The inter- and intra-observer variations of the spine doctors by both AP-LPR and CT method is shown in Table. 1. The mean kappa coefficient ranged from 0.874 to 0.969, indicating excellent agreement for each method. All of the suspected MA-LSTVs determined by AP-LPRs were verified to be real MA-LSTVs by CT-CRIs.

**Table 1:** The inter-and intra-observer variation for classification of MA-LSTV types by both AP-LPR method and CT method

Reading	Kappa (95% conf. limit)	
	LPR	CT
First inter-observer variation	0.893 (0.855–0.931)	0.921 (0.887–0.955)
Second inter-observer variation	0.880 (0.840–0.920)	0.874 (0.832–0.916)
intra-observer variation of observer 1	0.955 (0.929–0.981)	0.969 (0.947–0.991)
intra-observer variation of observer 2	0.939 (0.910–0.968)	0.952 (0.925–0.979)

In all, 39, 17, 2, 20 and 13 of the 91 cases were suspected to be type IIa, IIb, IIIa, IIIb and IV, respectively, based on AP-LPRs. Taking CT-CRIs as gold criteria, all of the suspected type IIIa and IIIb MA-LSTVs were verified to be unquestioned, while 9 of the 39 suspected type IIa, 9 and 3 of the 17 suspected type IIb, and 11 of the 13 suspected type IV were verified to be real type IIIa, IIIb, IV and IIIb, respectively. The accuracy rates of classification of type IIa, IIb, IIIa, IIIb and IV MA-LSTVs by AP-LPRs were 76.9, 29.4, 100, 100 and 15.4%, respectively. Generally, 32 MA-LSTVs were misclassified by AP-LPRs; the overall misclassification rate was 35.2% (Table. 2). The kappa values for agreement on the different MA-LSTV classification systems between the LPR and CT groups was 0.502 (0.446–0.561) for the first reading and 0.493 (0.434–0.552) for the second reading, namely, poor to moderate agreement.

**Table 2:** The MA-LSTV types suspected by AP-LPR and verified by CT-CRIs (*n* = 91)

Types	suspected by AP LPRs	Confirmed by CT-CRIs				
		IIa	IIb	IIIa	IIIb	IV
IIa	39	30	0	9	0	0
IIb	17	0	5	0	9	3
IIIa	2	0	0	2	0	0
IIIb	20	0	0	0	20	0
IV	13	0	0	0	11	2

The 32 misclassified cases could be further divided into 41 misclassified sides (9, 21 and 11 sides in suspected type IIa, IIb and IV MA-LSTVs, respectively). All misclassified sides were verified to be type IIIa transitional sides while misclassified as type IIa by AP-LPR. They were further divided into five situations.

**Fig. 1:**
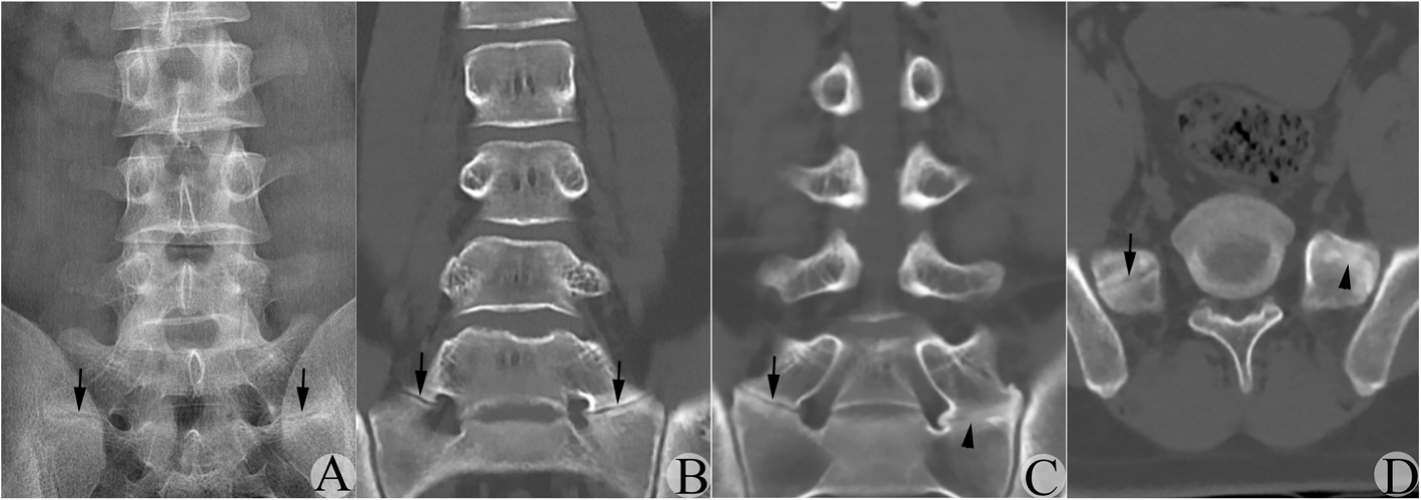
A suspected type IIb MA-LSTV judged by AP-LPR was verified to be type IV by CT-CRI. **a** AP-LPR image showing the suspected JLS between the TP of the MA-LSTV and sacrum bilaterally. Arrow indicates the suspected JLS. **b** CT-CRI showing the JLS was found in most CT-CRIs bilaterally. Arrow indicates the JLS. **c** CT-CRI showing the detected BUS on left side in only a few CT-CRIs through the posterior vertebral body. Arrow indicates the JLS; arrowhead indicates the BUS. **d** Transverse CT image verified the JLS on the right side, while the BUS was on the left side. Arrow indicates the JLS; arrowhead indicates the BUS

**Fig. 2:**
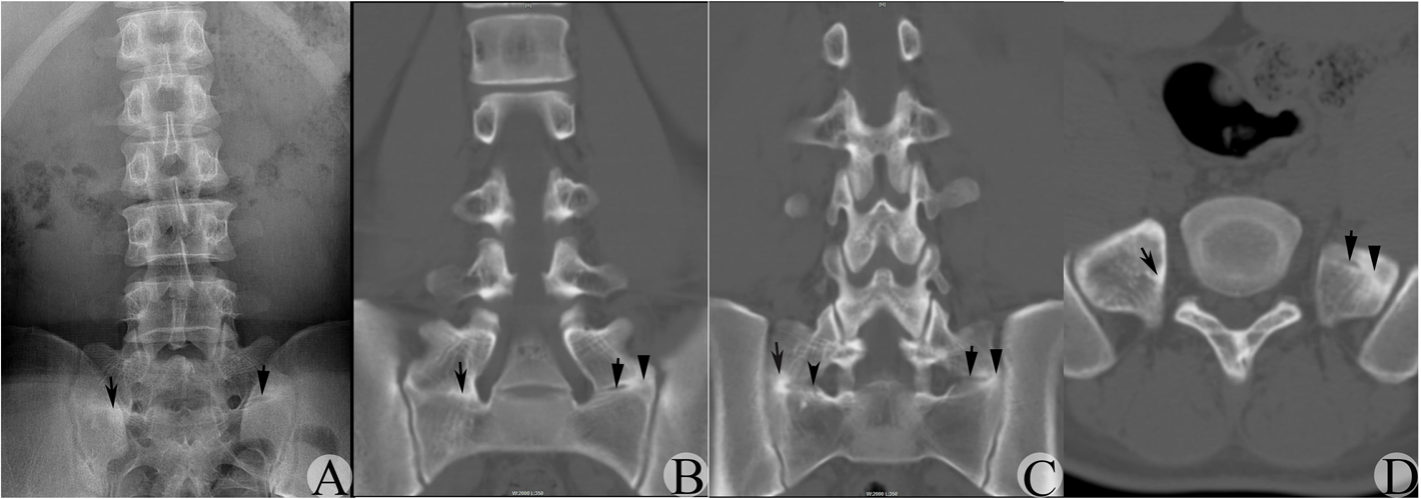
A suspected type IIb MA-LSTV judged by AP-LPR was verified to be type IIIb by CT-CRI. **a** AP-LPR image showing the suspected JLS existed bilaterally, and suspected JLS on the right side was narrower than that on the left side in the craniocaudal direction. Arrow indicates the suspected JLS on the left side; concave arrow indicates the suspected JLS on the right side. **b** CT-CRI showing the BUS was found in most CT-CRIs, while vague RSB was reserved at the medial region on the right side. Meanwhile, the intermittent JLS and BUS appeared simultaneously on the left side. Arrow, arrowhead and concave arrow indicates the JLS, BUS with RSB on the left side, and the BUS with RSB on the right side, respectively. **c** CT-CRI showing the detected JLS at the medial region in a few planes on the right side and the intermittent JLS and BUS appearing simultaneously at each plane on the left side. Arrow, arrowhead, concave arrow and concave arrowhead indicates the JLS and BUS on the left side, and the BUS and JLS on the right side, respectively. **d** Transverse CT images confirmed the JLS and BUS appeared in the same plane on the left side, and the BUS with RSB on the right side. Arrow, arrowhead and concave arrow indicates the JLS and BUS on the left side, and BUS with RSB on the right side, respectively

**Fig. 3:**
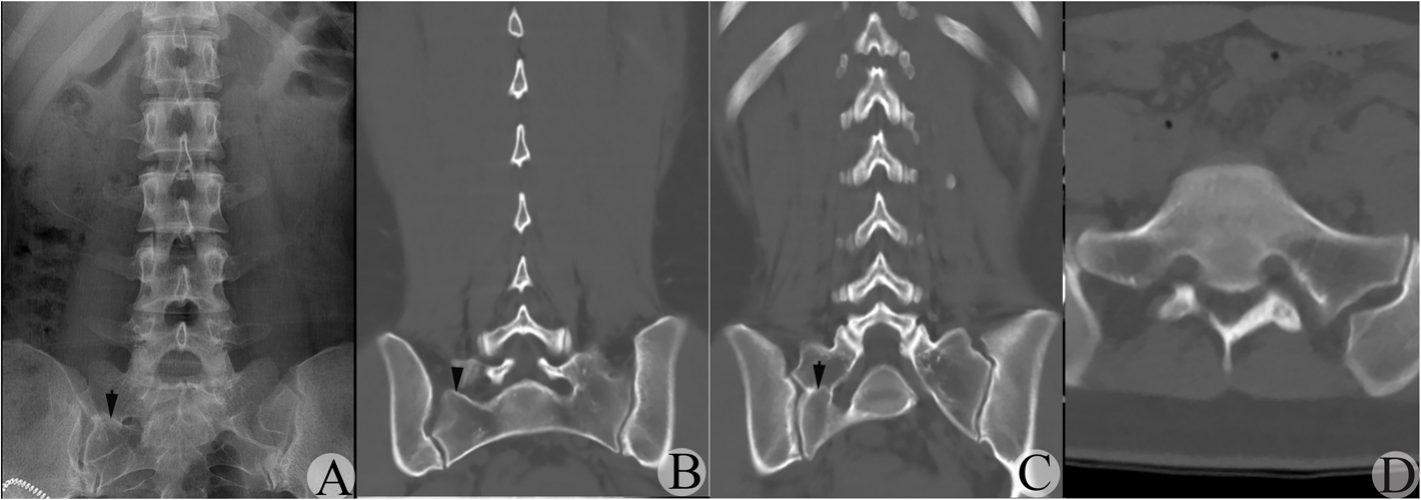
A suspected type IV MA-LSTV judged by AP-LPR was verified to be type IIIb by CT-CRI. **a** AP-LPR image showing the suspected JLS existed on the right side with narrowed connection width and RSB. Arrow indicates the suspected JLS. **b** CT-CRI showing the right TP of the MA-LSTV separated from the sacrum in some CT-CRIs through the vertebral arch planes. Arrowhead indicates the JLS with vague RSB on the right side. **c** CT-CRI showing BUS on the right side with RSB. Arrow indicates the BUS with RSB. **d** Transverse CT image confirmed bony connection bilaterally, while the connection region was much narrower and shorter on the right side compared to the left side

**Fig. 4:**
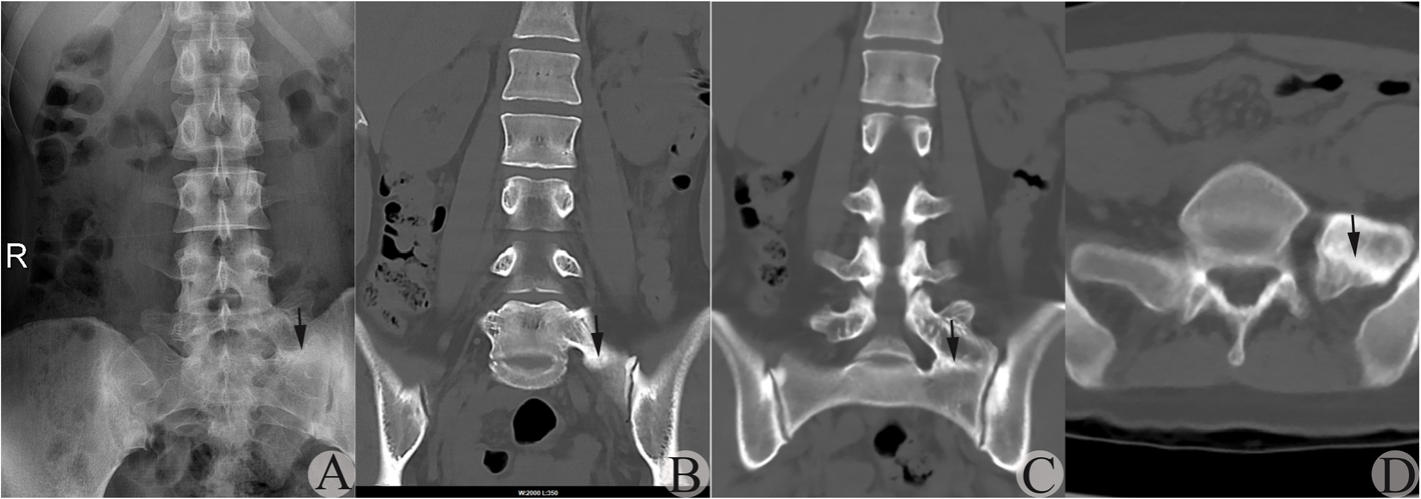
A suspected type IIa MA-LSTV judged by AP-LPR was verified to be type IIIa by CT-CRI. **a** AP-LPR image showing the suspected JLS on the left side. Arrow indicates the suspected JLS. **b** CT-CRI through vertebral body planes showing the suspected JLS to be RSB located at the bony fusion region. Arrow indicates the BUS with RSB. **c** CT-CRI through vertebral arch showing the suspected JLS to be RSB located at the bony fusion region; no JLS was detected. Arrow indicates the BUS with RSB. **d** Transverse CT image showing the BUS with RSB on the left side. Arrow indicates the BUS with RSB on the left side

1. Typical JLS was found in most CT-CRIs, while BUS was found in only a few CT-CRIs at the connection region. This happened in 9 sides (Fig. 1, left side).
2. Typical BUS was found in most CT-CRIs, while JLS appeared in only a few CT-CRIs. This happened in 8 sides (Fig. 2, right side).
3. JLS and BUS was found simultaneously in most CT-CRIs, with or without remnants of sclerotic band (RSB). This was found in 8 sides (Fig. 2, left side).
4. In 3 true type IIIb but suspected type IV cases, the real type IIIa but suspected IIa side was narrower than its contralateral side at the medial-to-lateral dimension with RSB (Fig. 3).
5. Thickened RSB made BUS region misinterpreted as JLS by AP-LPR. This happened in 13 sides (Fig. 4, left side).

**Fig. 5:**
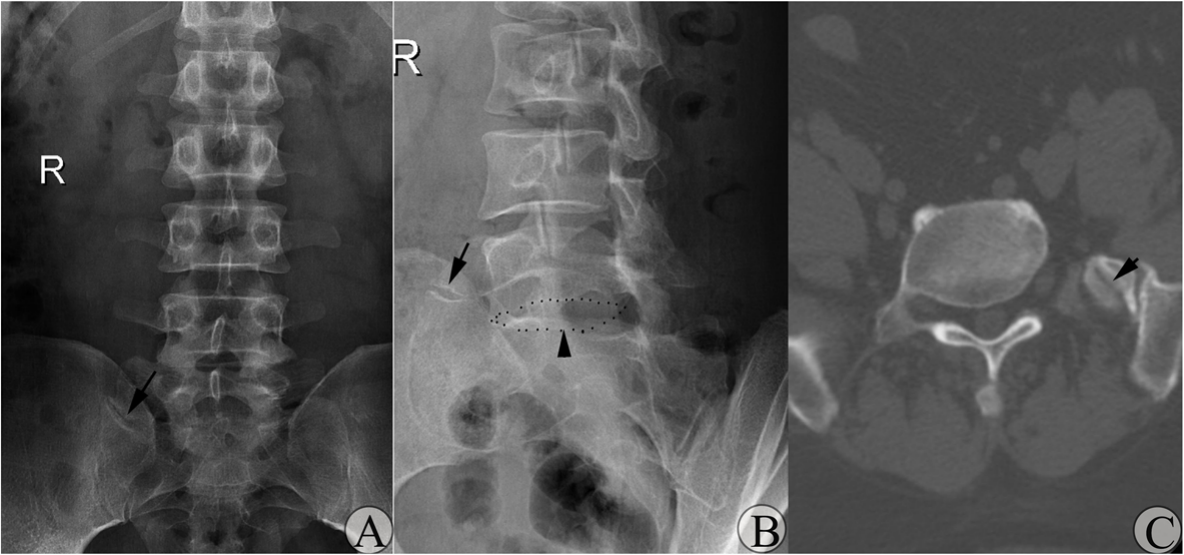
Variance of the orientation of the JLS seen in type IIa MA-LSTV. **a** AP-LPR image showing that the orientation of the JLS pointed upward when moving laterally instead of parallel to the horizontal line. Arrow indicates the JLS. **b** Oblique LPR showing orientation of the JBS was not parallel to the inferior endplate of the MA-LSTV. Arrow indicates the JBS. Arrowhead indicates the inferior endplate of the MA-LSTV. **c** Transaxial CT images showing the orientation of the JBS was not parallel to the coronal plane. Arrow indicates the JLS

According to the figures of our study, the JLS was not parallel to the horizontal line on AP-LPR (Fig. 5a), not parallel to the inferior endplate of the MA-LSTV on oblique LPR (Fig. 5b), and not parallel to the coronal plane on transaxial CT images (Fig. 5c).

## Discussion

LSTV was first regarded as a cause of low back pain by Bertolotti in 1917 [12]. Although there have been many studies on LSTV, many misunderstandings about its anatomical characteristics exist. Some anatomical landmarks suggested previously for determining spinal counts and LSTV were verified unreliable [5, 13]_,_ while they were still taken as criteria by some other studies [14, 15].

The Castellvi classification system was widely accepted for its simplicity. Prominent characteristics of each Castellvi type of MA-LSTV were initially extracted from the Ferguson view of LPR [3]. Considering that radiographic images form as a result of differing attenuation of the X-ray beam by various tissues within the patient, the characteristics of the concerned bony structures might be disturbed or covered by other surrounding structures. Recently, Farshad-Amacker et al. reported that coronal MRI is superior to standard AP-LPR in detecting and classifying of MA-LSTV [10]. However, MRI is inferior in detecting bony details compared with CT-CRIs. Moreover, coronal MRI is not routinely taken in routine clinical practice.

In our series, we found all suspected MA-LSTVs diagnosed by AP-LPR were verified to be true MA-LSTVs by CT-CRIs; however, AP-LPR could not classify MA-LSTV types with 100% accuracy. Type IIIb MA-LSTVs might be wrongly classified as type IV or IIb, while type IV or IIIa might be wrongly classified as type IIb and IIa, respectively. The agreement of classification between the LPR and CT groups was poor to moderate according to the statistical analysis.

What caused such misclassification? The following were our analysis.

1. BUS might not occupy the full space between the anomalous TP and sacrum at the type III transitional side

According to the Castellvi classification principle, if a continuous bone bridge forms between the TP of the MA-LSTV and the sacrum, the anomalous side belongs to a type III transition. One might take it for granted that bone bridge formation means that all space between the TP and sacrum should be fully occupied by continuous bone with disappearance of RSB. In reality, such a condition truly exists (Fig. 3, left side), but this only occurred in 48.8% (39/80) of type III sides in our series. Other situations might also exist. For example, only a limited percent of the space might reach bony fusion, while the remnant was occupied by JLS. Under such a condition, the abnormal side was a type III transition, but there was a high possibility that the side was misinterpreted as a type II side by AP-LPR. This could be further divided into 3 subcategories: (1) BUS only occupied a limited region, while JLS occupied most of the region (Fig. 1, left side); (2) BUS occupied most of the region, while JLS occupied a limited region (Fig. 2, right side); and (3) JLS and BUS shared almost equal space (Fig. 2, left side). These situations occurred in 31.3% (25/80) type III transitional sides and occupied 61.0% (25/41) of all misclassified sides.

2. Existence of RSB might lead to a type III transitional side being misdiagnosed as a type II transition side on AP-LPR

In some cases, complete fusion between the TP of the MA-LSTV and sacrum had been reached, while irregular RSB at the inferior boundary of the TP of the MA-LSTV and/or that at the superior boundary of the sacrum still existed. This might form a false image of anomalous articulation on AP-LPR, resulting in a type IIIa transition being misdiagnosed as a type IIa transition (Figs. 3 and 4). If discrepancy of width at the connection space existed at bilateral sides in a type IIIb case, there was a higher possibility that the narrower side with RSB was misdiagnosed as a type II transition. These situations occurred in 20.0% (16/80) of type III sides and occupied 39.0% (16/41) of misdiagnosed sides.

3. There might exist a progressive transformation process from type II transition to type III transition

In our series, we found type III transitional sides in many cases consisted of both JLS and BUS at the connection space instead of only occupied by BUS. The former occurred in 31.3% (25/80) of all type III sides, which was verified by CT-CRIs, while the distribution area or percentage of JLS or BUS varied in different cases. In the remaining 55 sides, 16 existing RSBs were found, which further occupied 19.8% (16/80) of type III transitional sides. Previously, we had taken it for granted that there only existed two clear categories at the space between the abnormal TP of the MA-LSTV and sacrum: one situation was that all space was replaced by continuous bone connection with complete disappearance of RSB and another was that all space was replaced by abnormal articulation structure. No transitional process existed between these two definite categories. When the reality revealed there existed the abovementioned situations, that is, both JLS and BUS co-existed at the connection space to different distribution or RSB left at the connection region, we hypothesized boldly that a type IIa transitional side might develop into a type IIIa transitional side under some special conditions. Initially, one type II transitional side might develop to the stage that the bony connection developed only in a limited region. Gradually, more JLS was replaced by bony bridge, until all connection space was replaced by bony bridge but with RSB remaining. At the final stage, RSB disappeared with complete rigid bony connection. Recently, Hou et al. reported one case who developed type IIIa MA-LSTV from type IIa following discectomy and fusion at the lumbosacral level [16]. This phenomenon partially supported our hypothesis. However, to verify this assumption, further follow-up on MA-LSTV with CT examination is needed. Additionally, if the hypothesis was true, the type of MA-LSTV should not be thought as congenital, but acquired, at least in some cases.

4. AP view instead of Ferguson view LPR was used for MA-LSTV type classification

In Castellvi’s original literature, a 30° angled AP view (Ferguson view) of the lumbar spine was regarded as the true AP of the lumbosacral joint, which could reach the purpose of removing the radiographic overlap of abnormal TP from MA-LSTV on the sacrum [3, 4, 10]. This viewpoint might be based on the following assumptions: (1) a 30° angled AP view of the lumbar spine indeed was the true AP of the lumbosacral joint; (2) the coronal plane of abnormal TP of the MA-LSTV was perpendicular to the inferior endplate of the MA-LSTV; (3) the inferior edge of the abnormal TP of the MA-LSTV was parallel to the inferior endplate of the MA-LSTV; and (4) the cleft between the TP of the MA-LSTV was parallel to the coronal plane observed in the transaxial view. However, the Ferguson view is not routinely taken as part of the standard radiographic assessment of the lumbar spine in clinical practice. As a retrospective study, we took AP instead of Ferguson view LPRs for MA-LSTV type classification. This might result in more misclassifications. However, according to our imaging data, we found the orientation of the JLS was irregular, neither parallel to the horizontal plane on AP view, nor to the coronal plane on transaxial view, nor to the endplate on oblique view (Fig. 5), which was theoretically impossible to offset by Ferguson view LPR. A recent study indicated that the Ferguson view had no superiority over the standard AP pelvis view for grading of sacroiliitis [17]. Further study might be needed to compare the AP view with the Ferguson view of the LPR in order to resolve whether one modality has a clear advantage for classification of MA-LSTV types.

Previously, conclusions of the relationship between various MA-LSTV types and their clinical significance were based on suspected MA-LSTV classification identified by LPRs, which might be questionable. The real relationship should be re-evaluated based on real MA-LSTV types identified by CT-CRIs.

## Conclusion

LPR was not reliable to identify MA-LSTV types according to the Castellvi classification principle. CT-CRIs could detect anatomic details at the connection region between the anomalous TP and sacrum, thus providing reliable LSTV type identification. Future studies on the relationship between MA-LSTV types and their clinical significance should take CT-CRI as a gold standard in the classification of MA-LSTVs.

## Data Availability

Data will be available upon request by the first and correspondence author Lisheng Hou.

## Abbreviations

LPR: Lumbar plain radiograph
AP-LPR: Anteroposterior view of lumbar plain radiograph
LSTV: Lumbosacral transitional vertebra
MA-LSTV: Lumbosacral transitional vertebra with morphological abnormality
NV-LSTV: Lumbosacral transitional vertebra with numerical variance
CT-CRIs: Coronal reconstructed CT images
JLS: Joint-like structure
BUS: Bony union structure
RSB: Remnants of sclerotic band
TP: Transverse process
